# Supplementary material for: Biomarker panels for improved risk prediction and enhanced biological insights in patients with atrial fibrillation
Source: Nat Commun. 2025 Jul 31;16:7042. doi: 10.1038/s41467-025-62218-7 (PMC12313968; doi:10.1038/s41467-025-62218-7)
Supplement: Supplementary file 1 — Supplementary Information [file 41467_2025_62218_MOESM1_ESM.pdf]

SUPPLEMENTARY MATERIAL

Biomarker panels for improved risk prediction and enhanced biological insights in patients with atrial fibrillation

Supplementary Table 1. Biomarkers and risk of composite of cardiovascular death, nonfatal ischemic stroke, nonfatal systemic embolism and nonfatal myocardial infarction

Supplementary Table 2. Biomarkers and risk of heart failure hospitalization

Supplementary Table 3. Biomarkers and risk of major bleeding

Supplementary Table 4. Biomarkers and risk of all strokes

Supplementary Table 5. Biomarkers and risk of ischemic stroke

Supplementary Table 6. Biomarkers and risk of myocardial infarction

Supplementary Table 7. Biomarkers and risk of cardiovascular death

Supplementary Table 8. Biomarkers and risk of all-cause death

Supplementary Table 9. Biomarkers and risk of any bleeding

Supplementary Table 10. Biomarkers and risk of clinically relevant non-major bleeding

Supplementary Table 11. Sensitivity analysis of biomarkers and risk of major bleeding in patients on oral anticoagulation (n=3,212)

Supplementary Table 12. Sensitivity analysis of biomarkers and risk of all strokes in patients on oral anticoagulation (n=3,212)

Supplementary Table 13. Sensitivity analysis of biomarkers and risk of ischemic stroke in patients on oral anticoagulation (n=3,212)

Supplementary Table 14. Discriminative ability of Cox and machine-learning models for outcomes with and without biomarkers

Supplementary Table 15. Discriminative ability of Cox and machine-learning models for outcomes with and without biomarkers of patients on oral anticoagulation (n=3,212)

Supplementary Table 16. Description of cohorts

Supplementary Table 17. Variables collected at baseline and included in the machine learning models

Supplementary Table 18. Description of biomarker measurements

Supplementary Table 19. Definition of major adverse cardiac events

Supplementary Figure 1. Spearman rank correlations of biomarkers

Supplementary Figure 2. Risk of adverse cardiovascular outcomes by biomarkers

Supplementary Figure 3. Associations between selected biomarkers and major bleeding, ischemic stroke and any stroke from combined Cox models of patients on oral anticoagulation (n=3,212)

Supplementary Figure 4. Relative importance of predictors from combined Cox models for major bleeding, ischemic stroke and any stroke of patients on oral anticoagulation (n=3,212)

Supplementary Figure 5. Discriminatory performance of clinical risk scores, biomarker-based models, and machine learning models for predicting stroke and major bleeding

Supplementary Figure 6. Predictive performance of Cox and machine learning models for major bleeding, ischemic stroke and any stroke with and without biomarkers of patients on oral anticoagulation (n=3,212)

Supplementary Figure 7. Flow diagram of the study

Supplementary Figure 8. Key pathophysiological pathways of the 12 selected biomarkers

Supplementary Figure 9. Missing pattern and multiple imputation of biomarkers

Supplementary Table 1. Biomarkers and risk of composite of cardiovascular death, nonfatal ischemic stroke, nonfatal systemic embolism and nonfatal myocardial infarction

	Age and sex adjusted model		Multivariable model		Combined model (backward selection)	
**Biomarker**	**HR (95% CI)***	**P value**	**HR (95% CI)**	**P value**	**HR (95% CI)**	**P value**
ANG-2	1.48 (1.39-1.58)	1.4x10^{-31}	1.35 (1.26-1.45)	3.9x10^{-17}	1.07 (0.98-1.17)	0.141978
eGFR	0.71 (0.65-0.77)	2.9x10^{-15}	0.79 (0.70-0.88)	1.5x10^{-5}	-	-
Cystatin C	1.43 (1.36-1.51)	6.2x10^{-44}	1.31 (1.23-1.40)	1.0x10^{-16}	-	-
D-dimer	1.23 (1.17-1.30)	4.4x10^{-16}	1.18 (1.12-1.24)	1.8x10^{-9}	1.09 (1.02-1.14)	0.010065
ALAT	0.87 (0.81-0.95)	0.00104	0.88 (0.81-0.95)	0.00146	0.87 (0.81-0.94)	0.000476
GDF-15	1.79 (1.67-1.92)	3.7x10^{-58}	1.58 (1.45-1.72)	5.1x10^{-25}	1.22 (1.11-1.35)	7.5x10^{-5}
Hs-CRP	1.21 (1.14-1.29)	1.9x10^{-9}	1.15 (1.08-1.23)	3.4x10^{-5}	-	-
IGFBP-7	1.54 (1.44-1.64)	1.2x10^{-37}	1.39 (1.29-1.50)	6.1x10^{-18}	-	-
IL-6	1.41 (1.34-1.49)	3.5x10^{-34}	1.31 (1.24-1.40)	5.9x10^{-19}	1.12 (1.04-1.21)	0.002527
NT-proBNP	1.91 (1.75-2.09)	7.4x10^{-46}	1.69 (1.53-1.86)	2.2x10^{-25}	1.30 (1.15-1.47)	2.1x10^{-5}
OPN	1.49 (1.41-1.58)	2.2x10^{-42}	1.39 (1.29-1.49)	3.3x10^{-18}	-	-
hsTropT	1.57 (1.48-1.66)	5.2x10^{-56}	1.47 (1.38-1.57)	6.5x10^{-30}	1.21 (1.11-1.32)	8.1x10^{-6}

*Hazard ratios (HR) and 95% confidence intervals (CI) were calculated using a Cox proportional hazards regression model. All HRs are standardized per 1-standard deviation (SD) increase in biomarker. P-values were derived from the Wald test, were two-sided and not adjusted for multiple comparisons. The multivariable models were adjusted for the following covariates: Age, sex, body mass index, current smoker status, systolic blood pressure, history of diabetes, prior stroke or transient ischemic attack (TIA), history of heart failure, chronic kidney disease, and coronary artery disease. The combined model further included all significant biomarkers after backward selection based on the Akaike Information Criterion (AIC).

Supplementary Table 2. Biomarkers and risk of heart failure hospitalization

	Age and sex adjusted model		Multivariable model		Combined model (backward selection)	
**Biomarker**	**HR (95% CI)***	**P value**	**HR (95% CI)**	**P value**	**HR (95% CI)**	**P value**
ANG-2	1.61 (1.51-1.72)	9.9x10^{-46}	1.41 (1.31-1.51)	4.2x10^{-22}	-	-
eGFR	0.68 (0.62-0.74)	1.8x10^{-17}	0.76 (0.68-0.84)	7.3x10^{-7}	-	-
Cystatin C	1.53 (1.46-1.61)	6.4x10^{-69}	1.34 (1.26-1.43)	8.9x10^{-19}	-	-
D-dimer	1.22 (1.15-1.28)	3.3x10^{-13}	1.13 (1.07-1.20)	1.1x10^{-5}	-	-
ALAT	0.92 (0.85-1.00)	0.06	0.93 (0.85-1.01)	0.07	0.91 (0.84-0.99)	0.03060
GDF-15	2.14 (1.99-2.30)	1.48x10^{-95}	1.86 (1.70-2.03)	4.8x10^{-42}	1.49 (1.33-1.66)	6.2x10^{-12}
Hs-CRP	1.28 (1.20-1.36)	1.1x10^{-13}	1.16 (1.08-1.24)	2.6x10^{-5}	-	-
IGFBP-7	1.76 (1.65-1.88)	3.3x10^{-65}	1.56 (1.44-1.68)	1.7x10^{-30}	1.12 (1.01-1.24)	0.03576
IL-6	1.44 (1.35-1.52)	1.7x10^{-33}	1.28 (1.20-1.37)	1.4x10^{-13}	-	-
NT-proBNP	2.36 (2.14-2.60)	1.5x10^{-67}	1.99 (1.79-2.21)	3.7x10^{-37}	1.55 (1.37-1.74)	1.9x10^{-12}
OPN	1.64 (1.55-1.74)	1.4x10^{-58}	1.40 (1.30-1.51)	6.7x10^{-18}	-	-
hsTropT	1.67 (1.58-1.76)	4.8x10^{-74}	1.52 (1.42-1.62)	5.8x10^{-33}	1.22 (1.11-1.34)	2.1x10^{-5}

*Hazard ratios (HR) and 95% confidence intervals (CI) were calculated using a Cox proportional hazards regression model. All HRs are standardized per 1-standard deviation (SD) increase in biomarker. P-values were derived from the Wald test, were two-sided and not adjusted for multiple comparisons. The multivariable models were adjusted for the following covariates: Age, sex, body mass index, current smoker status, systolic blood pressure, history of diabetes, prior stroke or transient ischemic attack (TIA), history of heart failure, chronic kidney disease, and coronary artery disease. The combined model further included all significant biomarkers after backward selection based on the Akaike Information Criterion (AIC).

Supplementary Table 3. Biomarkers and risk of major bleeding

	Age and sex adjusted model		Multivariable model		Combined model (backward selection)	
**Biomarker**	**HR (95% CI)***	**P value**	**HR (95% CI)**	**P value**	**HR (95% CI)**	**P value**
ANG-2	1.22 (1.11-1.34)	$3.8x10^{-5}$	1.16 (1.05-1.28)	0.00399	-	-
eGFR	0.80 (0.71-0.90)	0.000339	0.95 (0.82-1.11)	0.52	-	-
Cystatin C	1.32 (1.21-1.43)	$5.6x10^{-11}$	1.24 (1.12-1.37)	$5.6x10^{-5}$	-	-
D-dimer	1.12 (1.04-1.21)	0.0043	1.08 (0.99-1.18)	0.06	-	-
ALAT	0.93 (0.84-1.04)	0.20	0.95 (0.86-1.06)	0.34	-	-
GDF-15	1.53 (1.38-1.69)	$1.3x10^{-15}$	1.46 (1.29-1.65)	$1.4x10^{-9}$	1.30 (1.12-1.52)	0.000644
Hs-CRP	1.12 (1.03-1.23)	0.012	1.14 (0.99-1.20)	0.061	-	-
IGFBP-7	1.42 (1.29-1.56)	$5.3x10^{-13}$	1.32 (1.19-1.47)	$3.6x10^{-7}$	1.14 (1.01-1.30)	0.048090
IL-6	1.28 (1.18-1.40)	$7.4x10^{-9}$	1.23 (1.13-1.35)	$3.2x10^{-6}$	1.11 (1.01-1.23)	0.039263
NT-proBNP	1.37 (1.21-1.54)	$2.7x10^{-7}$	1.26 (1.11-1.43)	0.00043	-	-
OPN	1.37 (1.25-1.50)	$3.3x10^{-12}$	1.28 (1.16-1.43)	$3.6x10^{-6}$	-	-
hsTropT	1.39 (1.27-1.52)	$1.2x10^{-12}$	1.31 (1.19-1.45)	$1.2x10^{-7}$	1.16 (1.03-1.31)	0.014368

*Hazard ratios (HR) and 95% confidence intervals (CI) were calculated using a Cox proportional hazards regression model. All HRs are standardized per 1-standard deviation (SD) increase in biomarker. P-values were derived from the Wald test, were two-sided and not adjusted for multiple comparisons. The multivariable models were adjusted for the following covariates: Age, sex, body mass index, current smoker status, systolic blood pressure, history of diabetes, prior stroke or transient ischemic attack (TIA), history of heart failure, chronic kidney disease, and coronary artery disease. The combined model further included all significant biomarkers after backward selection based on the Akaike Information Criterion (AIC).

Supplementary Table 4. Biomarkers and risk of all strokes

	Age and sex adjusted model		Multivariable model		Combined model (backward selection)	
**Biomarker**	**HR (95% CI)***	**P value**	**HR (95% CI)**	**P value**	**HR (95% CI)**	**P value**
ANG-2	1.25 (1.10-1.42)	0.00055	1.20 (1.05-1.37)	0.0072	-	-
eGFR	0.98 (0.83-1.16)	0.83	1.07 (0.89-1.29)	0.49	-	-
Cystatin C	1.09 (0.95-1.25)	0.22	1.06 (0.89-1.25)	0.52	-	-
D-dimer	1.10 (0.98-1.22)	0.11	1.10 (0.98-1.24)	0.09	-	-
ALAT	0.93 (0.81-1.07)	0.31	0.92 (0.80-1.06)	0.26	0.90 (0.78-1.03)	0.12
GDF-15	1.16 (0.99-1.35)	0.051	1.09 (0.91-1.31)	0.33	-	-
Hs-CRP	1.09 (0.97-1.24)	0.15	1.09 (0.97-1.24)	0.16	-	-
IGFBP-7	1.14 (0.99-1.32)	0.07	1.08 (0.92-1.27)	0.35	-	-
IL-6	1.23 (1.09-1.38)	0.00054	1.20 (1.06-1.36)	0.004	1.16 (1.01-1.32)	0.0334
NT-proBNP	1.54 (1.31-1.80)	$1.0\text{x}10^{-7}$	1.53 (1.28-1.82)	$1.8\text{x}10^{-6}$	1.55 (1.29-1.87)	$4.1\text{x}10^{-6}$
OPN	1.08 (0.93-1.25)	0.31	1.04 (0.88-1.24)	0.63	0.86 (0.72-1.04)	0.12
hsTropT	1.23 (1.08-1.41)	0.0025	1.20 (1.03-1.39)	0.017	-	-

*Hazard ratios (HR) and 95% confidence intervals (CI) were calculated using a Cox proportional hazards regression model. All HRs are standardized per 1-standard deviation (SD) increase in biomarker. P-values were derived from the Wald test, were two-sided and not adjusted for multiple comparisons. The multivariable models were adjusted for the following covariates: Age, sex, body mass index, current smoker status, systolic blood pressure, history of diabetes, prior stroke or transient ischemic attack (TIA), history of heart failure, chronic kidney disease, and coronary artery disease. The combined model further included all significant biomarkers after backward selection based on the Akaike Information Criterion (AIC).

Supplementary Table 5. Biomarkers and risk of ischemic stroke

	Age and sex adjusted model		Multivariable model		Combined model (backward selection)	
**Biomarker**	**HR (95% CI)***	**P value**	**HR (95% CI)**	**P value**	**HR (95% CI)**	**P value**
ANG-2	1.27 (1.11-1.47)	0.00072	1.21 (1.04-1.41)	0.0116	-	-
eGFR	0.97 (0.80-1.18)	0.77	1.09 (0.86-1.33)	0.43	-	-
Cystatin C	1.08 (0.92-1.27)	0.34	1.02 (0.84-1.23)	0.88	-	-
D-dimer	1.07 (0.94-1.22)	0.29	1.08 (0.94-1.23)	0.28	-	-
ALAT	0.91 (0.78-1.06)	0.24	0.91 (0.78-1.06)	0.23	0.87 (0.74-1.02)	0.0891
GDF-15	1.18 (0.99-1.39)	0.06	1.06 (0.87-1.30)	0.56	-	-
Hs-CRP	1.09 (0.95-1.25)	0.24	1.08 (0.94-1.24)	0.29	-	-
IGFBP-7	1.13 (0.97-1.33)	0.13	1.04 (0.87-1.25)	0.65	-	-
IL-6	1.19 (1.04-1.36)	0.013	1.15 (0.99-1.32)	0.07	-	-
NT-proBNP	1.52 (1.27-1.82)	$3.6x10^{-6}$	1.49 (1.23-1.81)	$5.6x10^{-5}$	1.61 (1.30-1.98)	$8.4x10^{-6}$
OPN	1.02 (0.86-1.21)	0.81	0.95 (0.77-1.15)	0.58	0.82 (0.66-1.01)	0.0565
hsTropT	1.20 (1.03-1.40)	0.023	1.14 (0.95-1.36)	0.15	-	-

*Hazard ratios (HR) and 95% confidence intervals (CI) were calculated using a Cox proportional hazards regression model. All HRs are standardized per 1-standard deviation (SD) increase in biomarker. P-values were derived from the Wald test, were two-sided and not adjusted for multiple comparisons. The multivariable models were adjusted for the following covariates: Age, sex, body mass index, current smoker status, systolic blood pressure, history of diabetes, prior stroke or transient ischemic attack (TIA), history of heart failure, chronic kidney disease, and coronary artery disease. The combined model further included all significant biomarkers after backward selection based on the Akaike Information Criterion (AIC).

Supplementary Table 6. Biomarkers and risk of myocardial infarction

	Age and sex adjusted model		Multivariable model		Combined model (backward selection)	
**Biomarker**	**HR (95% CI)***	**P value**	**HR (95% CI)**	**P value**	**HR (95% CI)**	**P value**
ANG-2	1.27 (1.09-1.48)	0.0025	1.14 (0.97-1.34)	0.11	-	-
eGFR	0.82 (0.67-1.01)	0.06	0.84 (0.66-1.08)	0.17	-	-
Cystatin C	1.32 (1.15-1.51)	7.5×10^{-5}	1.14 (0.95-1.37)	0.15	-	-
D-dimer	1.15 (1.01-1.31)	0.036	1.09 (0.95-1.25)	0.22	-	-
ALAT	0.97 (0.82-1.15)	0.75	0.93 (0.79-1.11)	0.44	-	-
GDF-15	1.56 (1.33-1.84)	9.7×10^{-8}	1.29 (1.05-1.58)	0.0156	-	-
Hs-CRP	1.17 (1.02-1.36)	0.031	1.09 (0.94-1.27)	0.27	-	-
IGFBP-7	1.30 (1.10-1.53)	0.00179	1.13 (0.94-1.36)	0.21	-	-
IL-6	1.35 (1.19-1.54)	7.3×10^{-6}	1.24 (1.07-1.44)	0.0037	1.19 (1.02-1.39)	0.03068
NT-proBNP	1.46 (1.20-1.77)	0.00012	1.27 (1.03-1.57)	0.0257	-	-
OPN	1.35 (1.17-1.56)	5.3×10^{-5}	1.18 (0.98-1.42)	0.07	-	-
hsTropT	1.43 (1.24-1.64)	4.8×10^{-7}	1.29 (1.08-1.54)	0.0052	1.21 (1.01-1.45)	0.04806

*Hazard ratios (HR) and 95% confidence intervals (CI) were calculated using a Cox proportional hazards regression model. All HRs are standardized per 1-standard deviation (SD) increase in biomarker. P-values were derived from the Wald test, were two-sided and not adjusted for multiple comparisons. The multivariable models were adjusted for the following covariates: Age, sex, body mass index, current smoker status, systolic blood pressure, history of diabetes, prior stroke or transient ischemic attack (TIA), history of heart failure, chronic kidney disease, and coronary artery disease. The combined model further included all significant biomarkers after backward selection based on the Akaike Information Criterion (AIC).

Supplementary Table 7. Biomarkers and risk of cardiovascular death

	Age and sex adjusted model		Multivariable model		Combined model (backward selection)	
**Biomarker**	**HR (95% CI)***	**P value**	**HR (95% CI)**	**P value**	**HR (95% CI)**	**P value**
ANG-2	1.62 (1.50-1.74)	1.7×10^{-35}	1.44 (1.33-1.56)	3.5×10^{-19}	-	-
eGFR	0.64 (0.58-0.70)	8.8×10^{-20}	0.71 (0.62-0.80)	9.8×10^{-8}	-	-
Cystatin C	1.55 (1.47-1.63)	4.2×10^{-63}	1.41 (1.33-1.51)	1.6×10^{-26}	-	-
D-dimer	1.27 (1.20-1.34)	1.7×10^{-16}	1.19 (1.12-1.27)	6.8×10^{-9}	1.06 (0.99-1.14)	0.078298
ALAT	0.82 (0.74-0.90)	5.5×10^{-5}	0.83 (0.75-0.92)	0.00022	0.83 (0.76-0.92)	0.000150
GDF-15	2.14 (1.97-2.32)	7.6×10^{-75}	1.86 (1.69-2.06)	1.6×10^{-34}	1.35 (1.21-1.52)	1.6×10^{-7}
Hs-CRP	1.27 (1.18-1.37)	1.4×10^{-10}	1.19 (1.10-1.28)	1.1×10^{-5}	-	-
IGFBP-7	1.73 (1.61-1.86)	6.6×10^{-48}	1.55 (1.42-1.68)	1.9×10^{-24}	-	-
IL-6	1.51 (1.42-1.60)	1.3×10^{-38}	1.39 (1.29-1.48)	7.2×10^{-21}	1.16 (1.07-1.26)	0.000643
NT-proBNP	2.41 (2.16-2.70)	1.5×10^{-54}	2.08 (1.84-2.36)	8.9×10^{-31}	1.48 (1.30-1.70)	9.7×10^{-9}
OPN	1.67 (1.57-1.78)	1.6×10^{-60}	1.57 (1.44-1.70)	1.0×10^{-27}	-	-
hsTropT	1.76 (1.66-1.87)	5.0×10^{-74}	1.65 (1.54-1.78)	8.3×10^{-42}	1.30 (1.18-1.44)	7.5×10^{-8}

*Hazard ratios (HR) and 95% confidence intervals (CI) were calculated using a Cox proportional hazards regression model. All HRs are standardized per 1-standard deviation (SD) increase in biomarker. P-values were derived from the Wald test, were two-sided and not adjusted for multiple comparisons. The multivariable models were adjusted for the following covariates: Age, sex, body mass index, current smoker status, systolic blood pressure, history of diabetes, prior stroke or transient ischemic attack (TIA), history of heart failure, chronic kidney disease, and coronary artery disease. The combined model further included all significant biomarkers after backward selection based on the Akaike Information Criterion (AIC).

Supplementary Table 8. Biomarkers and risk of all-cause death

	**Age and sex adjusted model**		**Multivariable model**		**Combined model (backward selection)**	
**Biomarker**	**HR (95% CI)***	**P value**	**HR (95% CI)**	**P value**	**HR (95% CI)**	**P value**
ANG-2	1.55 (1.46-1.65)	5.6x10^{-45}	1.40 (1.31-1.49)	5.9x10^{-25}	-	-
eGFR	0.63 (0.58-0.68)	2.1x10^{-33}	0.67 (0.61-0.74)	8.7x10^{-16}	-	-
Cystatin C	1.56 (1.50-1.62)	1.1x10^{-102}	1.45 (1.38-1.53)	1.1x10^{-47}	-	-
D-dimer	1.27 (1.22-1.33)	4.7x10^{-26}	1.20 (1.14-1.26)	5.1x10^{-14}	1.07 (1.01-1.13)	0.018502
ALAT	0.86 (0.80-0.93)	0.00012	0.88 (0.81-0.95)	0.00088	0.88 (0.82-0.95)	0.001143
GDF-15	2.17 (2.04-2.32)	1.1x10^{-124}	1.95 (1.80-2.10)	2.1x10^{-63}	1.42 (1.28-1.57)	1.3x10^{-11}
Hs-CRP	1.33 (1.25-1.40)	1.3x10^{-22}	1.24 (1.17-1.32)	8.1x10^{-13}	-	-
IGFBP-7	1.76 (1.66-1.86)	2.2x10^{-80}	1.62 (1.52-1.73)	3.0x10^{-46}	1.12 (1.02-1.23)	0.014693
IL-6	1.52 (1.45-1.60)	7.8x10^{-64}	1.41 (1.34-1.49)	1.9x10^{-36}	1.19 (1.12-1.28)	1.5x10^{-7}
NT-proBNP	2.15 (1.96-2.34)	1.1x10^{-64}	1.88 (1.70-2.07)	4.9x10^{-37}	1.25 (1.12-1.39)	7.8x10^{-5}
OPN	1.68 (1.60-1.76)	1.3x10^{-99}	1.59 (1.50-1.69)	8.5x10^{-50}	1.02 (0.93-1.11)	0.72
hsTropT	1.71 (1.62-1.79)	1.5x10^{-97}	1.60 (1.50-1.69)	2.6x10^{-53}	1.22 (1.12-1.32)	3.2x10^{-6}

*Hazard ratios (HR) and 95% confidence intervals (CI) were calculated using a Cox proportional hazards regression model. All HRs are standardized per 1-standard deviation (SD) increase in biomarker. P-values were derived from the Wald test, were two-sided and not adjusted for multiple comparisons. The multivariable models were adjusted for the following covariates: Age, sex, body mass index, current smoker status, systolic blood pressure, history of diabetes, prior stroke or transient ischemic attack (TIA), history of heart failure, chronic kidney disease, and coronary artery disease. The combined model further included all significant biomarkers after backward selection based on the Akaike Information Criterion (AIC).

Supplementary Table 9. Biomarkers and risk of any bleeding

	Age and sex adjusted model		Multivariable model		Combined model (backward selection)	
**Biomarker**	**HR (95% CI)***	**P value**	**HR (95% CI)**	**P value**	**HR (95% CI)**	**P value**
ANG-2	1.20 (1.13-1.28)	4.0×10^{-8}	1.15 (1.07-1.23)	9.3×10^{-5}	-	-
eGFR	0.90 (0.82-0.98)	0.0170	0.94 (0.84-1.04)	0.23	-	-
Cystatin C	1.21 (1.14-1.29)	4.1×10^{-9}	1.16 (1.07-1.25)	0.00028	-	-
D-dimer	1.10 (1.04-1.17)	0.0011	1.08 (1.02-1.15)	0.01025	-	-
ALAT	0.97 (0.91-1.04)	0.46	0.97 (0.91-1.04)	0.41	-	-
GDF-15	1.40 (1.30-1.50)	3.4×10^{-19}	1.38 (1.27-1.51)	2.8×10^{-13}	1.25 (1.13-1.39)	1.7×10^{-5}
Hs-CRP	1.07 (1.00-1.14)	0.042	1.03 (0.97-1.11)	0.33	-	-
IGFBP-7	1.31 (1.22-1.40)	3.0×10^{-14}	1.25 (1.16-1.35)	6.6×10^{-9}	1.09 (0.99-1.20)	0.0716
IL-6	1.21 (1.13-1.28)	2.2×10^{-9}	1.16 (1.09-1.24)	5.2×10^{-6}	1.08 (1.01-1.16)	0.0339
NT-proBNP	1.29 (1.19-1.40)	4.2×10^{-10}	1.21 (1.12-1.32)	8.8×10^{-6}	1.06 (0.97-1.17)	0.21
OPN	1.24 (1.16-1.32)	5.4×10^{-10}	1.19 (1.10-1.29)	1.8×10^{-5}	-	-
hsTropT	1.25 (1.16-1.33)	1.6×10^{-10}	1.19 (1.10-1.28)	6.5×10^{-6}	-	-

*Hazard ratios (HR) and 95% confidence intervals (CI) were calculated using a Cox proportional hazards regression model. All HRs are standardized per 1-standard deviation (SD) increase in biomarker. P-values were derived from the Wald test, were two-sided and not adjusted for multiple comparisons. The multivariable models were adjusted for the following covariates: Age, sex, body mass index, current smoker status, systolic blood pressure, history of diabetes, prior stroke or transient ischemic attack (TIA), history of heart failure, chronic kidney disease, and coronary artery disease. The combined model further included all significant biomarkers after backward selection based on the Akaike Information Criterion (AIC).

Supplementary Table 10. Biomarkers and risk of clinically relevant non-major bleeding

	Age and sex adjusted model		Multivariable model		Combined model (backward selection)	
**Biomarker**	**HR (95% CI)***	**P value**	**HR (95% CI)**	**P value**	**HR (95% CI)**	**P value**
ANG-2	1.24 (1.15-1.35)	8.8x10^{-8}	1.20 (1.10-1.30)	2.7x10^{-5}	-	-
eGFR	0.97 (0.87-1.08)	0.53	0.95 (0.83-1.08)	0.43	-	-
Cystatin C	1.15 (1.05-1.25)	0.0015	1.11 (1.01-1.23)	0.03608	-	-
D-dimer	1.09 (1.01-1.17)	0.02	1.08 (1.00-1.16)	0.050	-	-
ALAT	1.02 (0.94-1.11)	0.67	1.01 (0.92-1.10)	0.86	-	-
GDF-15	1.33 (1.22-1.46)	5.1x10^{-10}	1.35 (1.22-1.51)	3.2x10^{-8}	1.26 (1.12-1.41)	0.000135
Hs-CRP	1.07 (0.99-1.16)	0.09	1.04 (0.96-1.13)	0.38	-	-
IGFBP-7	1.26 (1.15-1.37)	2.0x10^{-7}	1.24 (1.12-1.36)	1.1x10^{-5}	-	-
IL-6	1.19 (1.10-1.28)	1.1x10^{-5}	1.16 (1.07-1.25)	0.00055	1.07 (0.98-1.17)	0.13
NT-proBNP	1.31 (1.19-1.45)	4.6x10^{-8}	1.26 (1.13-1.40)	1.7x10^{-5}	1.15 (1.03-1.29)	0.013940
OPN	1.14 (1.04-1.25)	0.004	1.11 (1.00-1.23)	0.04421	-	-
hsTropT	1.16 (1.06-1.27)	0.00099	1.11 (1.01-1.23)	0.0344	-	-

*Hazard ratios (HR) and 95% confidence intervals (CI) were calculated using a Cox proportional hazards regression model. All HRs are standardized per 1-standard deviation (SD) increase in biomarker. P-values were derived from the Wald test, were two-sided and not adjusted for multiple comparisons. The multivariable models were adjusted for the following covariates: Age, sex, body mass index, current smoker status, systolic blood pressure, history of diabetes, prior stroke or transient ischemic attack (TIA), history of heart failure, chronic kidney disease, and coronary artery disease. The combined model further included all significant biomarkers after backward selection based on the Akaike Information Criterion (AIC).

Supplementary Table 11. Sensitivity analysis of biomarkers and risk of major bleeding in patients on oral anticoagulation (n=3,212)

	Age and sex adjusted model		Multivariable model		Combined model (backward selection)	
**Biomarker**	**HR (95% CI)***	**P value**	**HR (95% CI)**	**P value**	**HR (95% CI)**	**P value**
ANG-2	1.21 (1.09-1.34)	0.0002	1.15 (1.03-1.28)	0.009	-	-
eGFR	0.80 (0.70-0.91)	0.0009	0.96 (0.82-1.13)	0.66	-	-
Cystatin C	1.32 (1.21-1.44)	$9.2x10^{-10}$	1.24 (1.10-1.38)	0.0002	-	-
D-dimer	1.17 (1.07-1.28)	0.0004	1.13 (1.03-1.24)	0.009	-	-
ALAT	0.93 (0.83-1.04)	0.19	0.95 (0.85-1.06)	0.34	-	-
GDF-15	1.47 (1.32-1.65)	$1.1x10^{-11}$	1.40 (1.23-1.60)	$5.5x10^{-7}$	1.21 (1.03-1.41)	0.0217
Hs-CRP	1.14 (1.04-1.25)	0.007	1.11 (1.01-1.23)	0.03	-	-
IGFBP-7	1.39 (1.26-1.54)	$1.3x10^{-10}$	1.29 (1.15-1.45)	$1.1x10^{-5}$	1.11 (0.96-1.27)	0.14
IL-6	1.26 (1.15-1.38)	$1.1x10^{-6}$	1.22 (1.11-1.34)	$6.5x10^{-5}$	1.12 (1.00-1.24)	0.0439
NT-proBNP	1.31 (1.15-1.49)	$4.7x10^{-5}$	1.19 (1.04-1.37)	0.011	-	-
OPN	1.40 (1.27-1.54)	$3.3x10^{-12}$	1.32 (1.18-1.47)	$1.4x10^{-6}$	-	-
hsTropT	1.38 (1.25-1.53)	$2.3x10^{-10}$	1.31 (1.17-1.46)	$3.3x10^{-6}$	1.15 (1.00-1.31)	0.0411

*Hazard ratios (HR) and 95% confidence intervals (CI) were calculated using a Cox proportional hazards regression model. All HRs are standardized per 1-standard deviation (SD) increase in biomarker. P-values were derived from the Wald test, were two-sided and not adjusted for multiple comparisons. The multivariable models were adjusted for the following covariates: Age, sex, body mass index, current smoker status, systolic blood pressure, history of diabetes, prior stroke or transient ischemic attack (TIA), history of heart failure, chronic kidney disease, and coronary artery disease. The combined model further included all significant biomarkers after backward selection based on the Akaike Information Criterion (AIC).

Supplementary Table 12. Sensitivity analysis of biomarkers and risk of all strokes in patients on oral anticoagulation (n=3,212)

	Age and sex adjusted model		Multivariable model		Combined model (backward selection)	
**Biomarker**	**HR (95% CI)***	**P value**	**HR (95% CI)**	**P value**	**HR (95% CI)**	**P value**
ANG-2	1.27 (1.11-1.46)	0.0004	1.22 (1.06-1.41)	0.005	-	-
eGFR	0.98 (0.82-1.18)	0.86	1.07 (0.88-1.30)	0.52	-	-
Cystatin C	1.11 (0.96-1.29)	0.15	1.08 (0.90-1.29)	0.40	-	-
D-dimer	1.13 (0.99-1.28)	0.07	1.12 (0.98-1.28)	0.09	-	-
ALAT	0.87 (0.75-1.02)	0.08	0.87 (0.74-1.01)	0.07	0.85 (0.73-0.98)	0.030
GDF-15	1.16 (0.98-1.37)	0.08	1.08 (0.89-1.31)	0.44	-	-
Hs-CRP	1.04 (0.91-1.19)	0.58	1.03 (0.90-1.18)	0.67	-	-
IGFBP-7	1.16 (0.99-1.35)	0.06	1.09 (0.92-1.29)	0.31	-	-
IL-6	1.21 (1.06-1.37)	0.0047	1.17 (1.02-1.34)	0.0272	1.11 (0.95-1.29)	0.19
NT-proBNP	1.56 (1.33-1.90)	2.6×10^{-7}	1.59 (1.31-1.93)	2.8×10^{-6}	1.62 (1.32-2.00)	5.1×10^{-6}
OPN	1.11 (0.96-1.30)	0.17	1.08 (0.90-1.30)	0.40	0.89 (0.73-1.09)	0.25
hsTropT	1.30 (1.13-1.50)	0.0003	1.29 (1.09-1.51)	0.00218	-	-

*Hazard ratios (HR) and 95% confidence intervals (CI) were calculated using a Cox proportional hazards regression model. All HRs are standardized per 1-standard deviation (SD) increase in biomarker. P-values were derived from the Wald test, were two-sided and not adjusted for multiple comparisons. The multivariable models were adjusted for the following covariates: Age, sex, body mass index, current smoker status, systolic blood pressure, history of diabetes, prior stroke or transient ischemic attack (TIA), history of heart failure, chronic kidney disease, and coronary artery disease. The combined model further included all significant biomarkers after backward selection based on the Akaike Information Criterion (AIC).

Supplementary Table 13. Sensitivity analysis of biomarkers and risk of ischemic stroke in patients on oral anticoagulation (n=3,212)

	Age and sex adjusted model		Multivariable model		Combined model (backward selection)	
**Biomarker**	**HR (95% CI)***	**P value**	**HR (95% CI)**	**P value**	**HR (95% CI)**	**P value**
ANG-2	1.33 (1.15-1.55)	0.00015	1.27 (1.08-1.49)	0.00326	-	-
eGFR	0.98 (0.80-1.20)	0.86	1.09 (0.88-1.35)	0.41	-	-
Cystatin C	1.13 (0.95-1.33)	0.16	1.06 (0.86-1.29)	0.60	-	-
D-dimer	1.12 (0.97-1.30)	0.12	1.12 (0.96-1.30)	0.16	-	-
ALAT	0.87 (0.73-1.03)	0.10	0.86 (0.73-1.02)	0.09	0.83 (0.70-0.99)	0.0338
GDF-15	1.21 (1.01-1.45)	0.037	1.09 (0.87-1.35)	0.46	-	-
Hs-CRP	1.07 (0.92-1.25)	0.35	1.06 (0.91-1.23)	0.48	-	-
IGFBP-7	1.16 (0.98-1.38)	0.09	1.06 (0.88-1.29)	0.53	-	-
IL-6	1.21 (1.04-1.40)	0.012	1.15 (0.99-1.35)	0.07	-	-
NT-proBNP	1.62 (1.33-1.98)	1.8×10^{-6}	1.61 (1.29-2.00)	2.2×10^{-5}	1.73 (1.37-2.19)	5.0×10^{-6}
OPN	1.08 (0.90-1.29)	0.43	0.99 (0.81-1.23)	0.98	0.84 (0.67-1.04)	0.11
hsTropT	1.31 (1.11-1.54)	0.00115	1.26 (1.05-1.52)	0.0140	-	-

*Hazard ratios (HR) and 95% confidence intervals (CI) were calculated using a Cox proportional hazards regression model. All HRs are standardized per 1-standard deviation (SD) increase in biomarker. P-values were derived from the Wald test, were two-sided and not adjusted for multiple comparisons. The multivariable models were adjusted for the following covariates: Age, sex, body mass index, current smoker status, systolic blood pressure, history of diabetes, prior stroke or transient ischemic attack (TIA), history of heart failure, chronic kidney disease, and coronary artery disease. The combined model further included all significant biomarkers after backward selection based on the Akaike Information Criterion (AIC).

Supplementary Table 14. Discriminative ability of Cox and machine-learning models for outcomes with and without biomarkers

Outcomes	Model	AUC_{Base} (95% CI)	$AUC_{Base+biomakers}$ (95% CI)	P value
Composite	Combined Cox model	0.74 (0.72-0.76)	0.77 (0.75-0.79)	2.6×10^{-8}
	LASSO	0.77 (0.75-0.79)	0.78 (0.76-0.80)	0.3235
	Random forest	0.74 (0.72-0.76)	0.75 (0.73-0.78)	0.02796
	XGBoost	0.95 (0.94-0.96)	0.97 (0.96-0.96)	0.0007345
HF hospitalization	Combined Cox model	0.77 (0.75-0.78)	0.80 (0.79-0.82)	5.5×10^{-10}
	LASSO	0.80 (0.78-0.82)	0.83 (0.81-0.84)	0.04671
	Random forest	0.77 (0.74-0.79)	0.80 (0.78-0.82)	0.0002564
	XGBoost	0.96 (0.95-0.97)	0.98 (0.97-0.98)	5.0×10^{-6}
Major bleeding	Combined Cox model	0.67 (0.64-0.69)	0.68 (0.66-0.71)	0.01352
	LASSO	0.69 (0.66-0.72)	0.70 (0.67-0.73)	0.5016
	Random forest	0.63 (0.60-0.66)	0.65 (0.62-0.68)	0.1015
	XGBoost	0.94 (0.93-0.96)	0.97 (0.96-0.98)	8.8×10^{-5}
Ischemic stroke	Combined Cox model	0.65 (0.61-0.69)	0.67 (0.63-0.71)	0.03113
	LASSO	0.68 (0.63-0.72)	0.69 (0.64-0.73)	0.7793
	Random forest	0.59 (0.54-0.64)	0.59 (0.54-0.64)	0.8381
	XGBoost	0.95 (0.93-0.97)	0.98 (0.97-0.99)	5.6×10^{-5}
Any stroke	Combined Cox model	0.66 (0.63-0.70)	0.68 (0.65-0.72)	0.02533
	LASSO	0.69 (0.65-0.73)	0.70 (0.66-0.74)	0.8273
	Random forest	0.61 (0.57-0.66)	0.61 (0.57-0.65)	0.8254
	XGBoost	0.95 (0.94-0.96)	0.97 (0.96-0.98)	0.01199
MI	Combined Cox model	0.70 (0.66-0.74)	0.70 (0.66-0.74)	0.943

	LASSO	0.75 (0.71-0.80)	0.74 (0.70-0.79)	0.7338
	Random forest	0.68 (0.63-0.72)	0.62 (0.57-0.67)	0.0009897
	XGBoost	0.97 (0.96-0.99)	0.99 (0.98-0.99)	0.02063
CV death	Combined Cox model	0.79 (0.77-0.81)	0.83 (0.81-0.85)	$5.6x10^{-14}$
	LASSO	0.82 (0.80-0.85)	0.85 (0.83-0.87)	0.09099
	Random forest	0.79 (0.77-0.82)	0.82 (0.80-0.85)	0.0001046
	XGBoost	0.97 (0.96-0.98)	0.98 (0.98-0.99)	0.0004972
All-cause death	Combined Cox model	0.80 (0.78-0.81)	0.84 (0.83-0.86)	$2.2x10^{-16}$
	LASSO	0.82 (0.80-0.84)	0.85 (0.83-0.87)	0.01385
	Random forest	0.80 (0.78-0.82)	0.83 (0.81-0.85)	$1.9x10^{-7}$
	XGBoost	0.95 (0.94-0.96)	0.98 (0.97-0.98)	$5.6x10^{-10}$
Any bleeding	Combined Cox model	0.64 (0.62-0.66)	0.65 (0.63-0.67)	0.07765
	LASSO	0.66 (0.64-0.69)	0.68 (0.66-0.71)	0.189
	Random forest	0.63 (0.61-0.66)	0.64 (0.62-0.67)	0.2455
	XGBoost	0.92 (0.91-0.94)	0.92 (0.91-0.94)	0.8432
CRNMB	Combined Cox model	0.61 (0.59-0.64)	0.62 (0.60-0.64)	0.2217
	LASSO	0.66 (0.63-0.68)	0.66 (0.63-0.69)	0.8349
	Random forest	0.60 (0.58-0.63)	0.63 (0.60-0.65)	0.05211
	XGBoost	0.94 (0.93-0.96)	0.96 (0.95-0.97)	0.007685

Area Under the Receiver Operating Characteristic curve (AUC) and 95% confidence interval (CI) were calculated for Cox proportional hazards models and machine learning models, with and without biomarkers, for each specified outcome. P-values were derived from a two-sided DeLong's test. No adjustments for multiple comparisons were applied to these p-values. Cox base model included age, sex, body mass index, current smoker, systolic blood pressure, history of diabetes, prior stroke or TIA, history of heart failure, chronic kidney disease and coronary artery disease. Base models from machine learning models (LASSO, Random forest, XGBoost) included all variables listed in the Supplementary Table 2.

Supplementary Table 15. Discriminative ability of Cox and machine-learning models for outcomes with and without biomarkers of patients on oral anticoagulation (n=3,212)

Outcomes	Model	AUC_{Base} (95% CI)	$AUC_{Base+biomakers}$ (95% CI)	P value
Major bleeding	Combined Cox model	0.65 (0.62-0.68)	0.67 (0.65-0.70)	0.01212
	LASSO	0.67 (0.64-0.71)	0.68 (0.64-0.71)	0.8446
	Random forest	0.61 (0.57-0.65)	0.63 (0.59-0.66)	0.2568
	XGBoost	0.95 (0.93-0.96)	0.98 (0.97-0.99)	0.0001649
Ischemic stroke	Combined Cox model	0.65 (0.61-0.69)	0.69 (0.65-0.73)	0.005113
	LASSO	0.69 (0.64-0.73)	0.70 (0.65-0.75)	0.6453
	Random forest	0.58 (0.53-0.64)	0.59 (0.54-0.64)	0.6897
	XGBoost	0.97 (0.96-0.99)	0.98 (0.96-0.99)	0.5465
Any stroke	Combined Cox model	0.66 (0.62-0.70)	0.69 (0.66-0.73)	0.002828
	LASSO	0.69 (0.65-0.74)	0.71 (0.66-0.75)	0.7215
	Random forest	0.60 (0.55-0.65)	0.61 (0.56-0.65)	0.8688
	XGBoost	0.96 (0.95-0.98)	0.99 (0.98-0.99)	0.0003617

Area Under the Receiver Operating Characteristic curve (AUC) and 95% confidence interval (CI) were calculated for Cox proportional hazards models and machine learning models, with and without biomarkers, for each specified outcome. P-values were derived from a two-sided DeLong's test. No adjustments for multiple comparisons were applied to these p-values. Cox base model included age, sex, body mass index, current smoker, systolic blood pressure, history of diabetes, prior stroke or TIA, history of heart failure, chronic kidney disease and coronary artery disease. Base models from machine learning models (LASSO, Random forest, XGBoost) included all variables listed in the Supplementary Table 2.

Supplementary Table 16. Description of cohorts

BEAT-AF	Swiss-AF
N=1,546	N=2,415
**Inclusion criteria**	
Documented (by electrocardiogram [ECG], rhythm strip or device interrogation) paroxysmal AF, persistent AF or permanent AF	Documented (by ECG, rhythm strip or device interrogation) paroxysmal AF, persistent AF or permanent AF
	Age ≥65 years
**Exclusion criteria**	
Any acute illness within the last 4 weeks. These patients could be enrolled after stabilization of their acute episode	Any acute illness within the last 4 weeks. These patients could be enrolled after stabilization of their acute episode
Patients who exclusively have short episodes of reversible forms of AF (e.g. AF post cardiac surgery, thyrotoxicosis)	Patients who exclusively have short episodes of reversible forms of AF (e.g. AF post cardiac surgery, thyrotoxicosis)
Inability to sign informed consent	Inability to sign informed consent
	Participating in BEAT-AF
**Patient recruitment periods**	
January 2010 - April 2014	January 2014 - September 2017
**Participating Study Centers**	
University Hospital Basel	University Hospital Basel
Cantonal Hospital St. Gallen	Cantonal Hospital St. Gallen
Cantonal Hospital Bellinzona	Cantonal Hospital Bellinzona
Cantonal Hospital Lugano	Cantonal Hospital Lugano
Hospital Rheinfelden	University Hospital Lausanne
University Hospital Zürich	Cantonal Hospital Lucerne
University Hospital Lausanne	University Hospital Geneva
Cantonal Hospital Lucerne	Cantonal Hospital Baden
University Hospital Geneva	University Hospital Berne
	Cardiocentro Ticino, Lugano
	Cantonal Hospital Fribourg
	Hospital St. Anna, Lucerne
	Stadtspital Triemli, Zürich

1

Supplementary Table 17. Variables collected at baseline and included in the machine learning models

Variable	Description
Age	years
Sex	Male, female
BMI	kg/m^2
Smoking	Yes=active or No=Past or never
Systolic blood pressure	mmHg
Diabetes	Yes or No
Prior stroke or TIA	Yes or No
Heart failure	Yes or No
Renal failure	Yes or No
Coronary artery disease	Yes or No
Study center	University Hospital Basel and Basel University, University Hospital Bern, Stadtspital Triemli Zurich, Kantonspital Baden, Cardiocentro Lugano, Kantonsspital St. Gallen, Hôpital Cantonal Fribourg, Luzerner Kantonsspital, Ente Ospedaliero Cantonale Lugano, University Hospital Geneva, University Hospital Lausanne, Bürgerspital Solothurn, Ente Ospedaliero Cantonale Bellinzona, University of Zurich/University Hospital Zurich, Hospital Rheinfelden, Hospital St. Anna, Lucerne
Ethnicity	Central Europe, Southern Europe, Northern Europe, Eastern Europe, Central/South America, North America, Other
AF type	Paroxysmal, persistent, permanent
Beer drinker	>6/day, 4-5/day, 2-3/day, 1/day, 5-6/week, 2-4/week, 1/week, 1-3/month, never or <1/month
Red wine drinker	>6/day, 4-5/day, 2-3/day, 1/day, 5-6/week, 2-4/week, 1/week, 1-3/month, never or <1/month
White wine drinker	>6/day, 4-5/day, 2-3/day, 1/day, 5-6/week, 2-4/week, 1/week, 1-3/month, never or <1/month
Liquor drinker	>6/day, 4-5/day, 2-3/day, 1/day, 5-6/week, 2-4/week, 1/week, 1-3/month, never or <1/month
Atrial fibrillation duration	Permanent, days, hours, minutes, no more
Nr. of atrial fibrillation episodes	Permanent, >1/week, 1/week, <1/week but >1/month, <1/month, no more
Atrial flutter	Yes or No
Aspirin	Yes or No
Antiplatelet therapy	Yes or No
Ticagrelor	Yes or No
Ilicit drugs	Yes or No
Cancer	Yes or No

VTE	Yes or No
Paternal AF history	Father with a history of AF, Yes or No
Brother AF history	Brother with a history of AF , Yes or No
Maternal AF history	Mother with a history of AF, Yes or No
Sister AF history	Sister with a history of AF , Yes or No
Family history of hypertension	Yes or No
Family history of diabetes	Yes or No
Family history of obesity	Yes or No
Family history of coronary artery disease	Yes or No
Rhythm on ECG at inclusion	Atrial fibrillation, atrial flutter, sinus rhythm, other
Hyperthyroidism	Yes or No
Hypothyroidism	Yes or No
Prior CABG	Yes or No
Height	cm
Weight	kg
Heart rate	bpm
Diastolic blood pressure	mmHg
Hypertension	Yes or No
Peripheral artery disease	Yes or No
Prior myocardial infarction	Yes or No
Systemic embolism	Yes or No
Prior major bleeding	Yes or No

1

Supplementary Table 18. Description of biomarker measurements

Biomarker	Methods
D-dimer	D-dimer values were analyzed via a commercial Tina-quant D-Dimer Gen.2 test (Roche Diagnostics, Mannheim, Germany) on a cobas C311 analyzer (Roche) according to the manufacturer's instructions. Values are provided in ug/mL. The limit of detection (LoD) was 0.150 ug/ml.
IL-6	The commercial Elecsys IL-6 assay (cobas e601, Roche Diagnostics, Mannheim, Germany) was used to measure plasma levels of IL-6. The minimal determined IL-6 was 1.5 pg/ml (LoD)
NT-proBNP	NT-proBNP was determined using the commercial Roche Elecsys proBNP II IVD on a cobas e601 (measuring range 10–35000ng/L) with a coefficient of variation of 2.45% for the lower control measured (mean 133.1ng/L). The assays applied are based on the Elecsys electro-chemiluminescence technology.
IGFBP-7	IGFBP-7 levels were measured with a precommercial Elecsys assay using an automated cobas electrochemiluminescence immunoassay analyzer e601 (Roche Diagnostics, Mannheim, Germany) For IGFBP-7, the limit of detection was 0.01 ng/mL, and the within-run precision coefficient of variation was 2%.
Cystatin C	Cystatin C levels were determined with the commercial Tina-quant Cystatin C Gen.2 assay (cobas c 311; Roche Diagnostics, Mannheim, Germany) with a lower limit of detection of 0.4 mg/L.
Hs-CRP	The commercial Cardiac C-Reactive Protein (Latex) High Sensitive assay was used (cobas c 311, Roche Diagnostics, Mannheim, Germany) to measure plasma levels of hs-CRP. The lower limit of detection was 0.15 mg/L.
OPN	OPN levels were measured with a precommercial Elecsys assay using an automated cobas electrochemiluminescence immunoassay analyzer e601 (Roche Diagnostics, Mannheim, Germany). For OPN, the limit of detection was 0.01 ng/mL.
GDF-15	Growth differentiation factor-15 (GDF-15), was determined by commercially available Elecsys assay on a e601 (Elecsys®; Roche Diagnostics, Mannheim, Germany). LoD was 22pg/mL.

hsTropT	Levels of hs-Troponin T (TNTHS) were determined by commercially available Elecsys assay on a e601 (oche Diagnostics, Mannheim, Germany). LoD was 3 ng/L.
ANG-2	Ang2 levels were measured with a precommercial Elecsys assay using an automated cobas electrochemiluminescence immunoassay analyzer e601 (Roche Diagnostics, Mannheim, Germany). LoD was 0.03 ng/mL.
ALAT	ALAT levels were determined with the commercially available ALTL assay (cobas c 311; Roche Diagnostics, Mannheim, Germany) with a LoD of 5 U/L (0.08 µkat/L).

Supplementary Table 19. Definition of major adverse cardiac events

Major bleeding
Major bleeding is defined according to the International Society on Thrombosis and Haemostasis criteria, as clinically overt bleeding with a fatal outcome, a reduction in haemoglobin level of ≥20 g/l within 7 days, transfusion of at least two units of blood, or symptomatic bleeding in a critical area or organ (intracranial, intraspinal, intraocular, pericardial, intra-articular, intramuscular with compartment syndrome, retroperitoneal).[1]
Clinically relevant non-major bleeding
CRNMB is defined as bleeding not fulfilling the ISTH criteria, but that was clinically overt and led to hospitalization, change of antithrombotic therapy, or necessitated a medical or surgical intervention.[2]
Stroke
Stroke is defined as an acute focal neurological deficit of vascular origin, with evidence of focal infarction confirmed by imaging (computed tomography or cMRI) or autopsy. Stroke is categorised as ischaemic, haemorrhagic or of unknown cause (based on computed tomography, cMRI, autopsy and other ancillary investigations). Ischaemic strokes are further classified according to the TOAST classification.[3] Fatal stroke is defined as death from any cause within 30 days after stroke.
Myocardial infarction
Myocardial infarction is defined as rise and/or fall of cardiac troponin with at least one value above the 99th percentile of the upper reference limit in a clinical setting consistent with myocardial ischaemia, and with at least one of the following: symptoms of ischaemia, new significant ST-T changes or new left bundle-branch block on ECG, development of pathological Q waves in the ECG, imaging evidence of new loss of viable myocardium or new regional wall motion abnormality, identification of an intracoronary thrombus by angiography or autopsy.[4]
Death
The cause of death is classified as being of cardiac, stroke, any cardiovascular, bleeding, non-cardiovascular or unknown aetiology.

Supplementary Figure 1. Spearman rank correlations of biomarkers

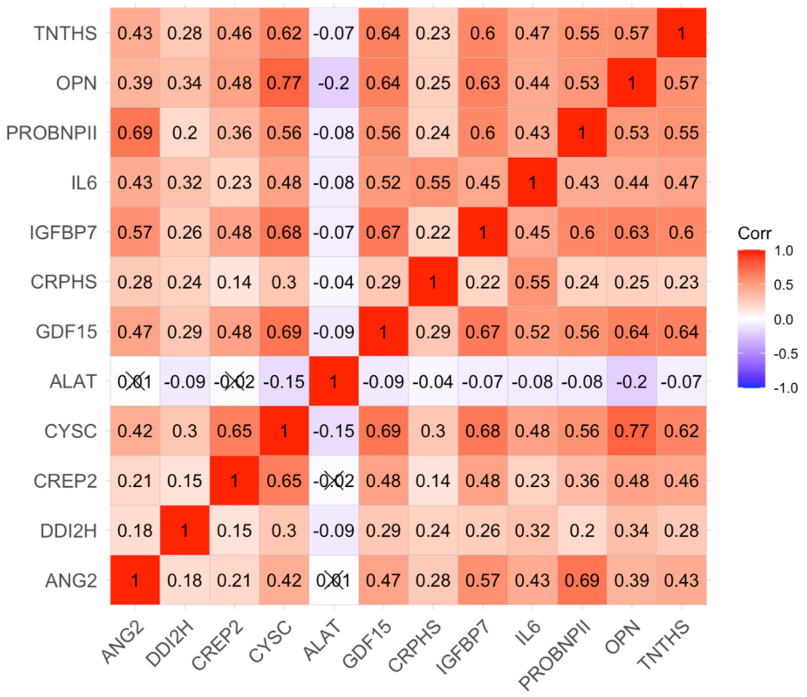

This figure shows the correlations between measured biomarkers. The size and color intensity of each circle correspond to the strength and direction of the correlation. Data were derived from N=3,817 AF patients.

Abbreviations: ANG2=Angiopoetin-2; DDI2H=D-dimer; CREP2=Creatinine; CYCS=Cystatin C; ALAT=Alanine aminotransferase; GDF-15=Growth differentiation factor-15; CRPHS= C-reactive protein high sensitive; IGFBP7= Insulin-like growth factor-binding protein-7; IL6= Interleukin-6; PROBNPII=NT-proBNP; OPN=Osteopontin; TNTHS=Troponin T high sensitive.

Supplementary Figure 2. Risk of adverse cardiovascular outcomes by biomarkers

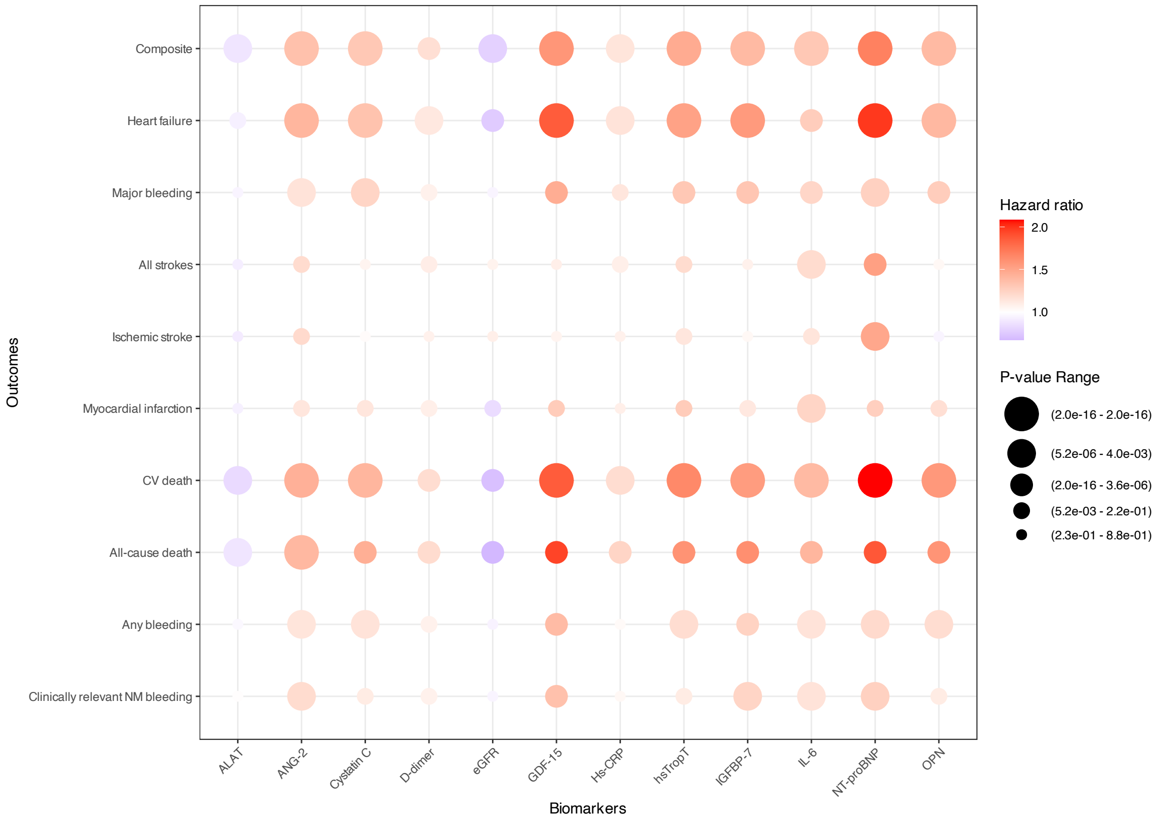

This figure shows the risk of adverse cardiovascular outcomes by the 12 measured biomarkers. Risk estimates are from multivariable models adjusted for age, sex, body mass index, current smoker, systolic blood pressure, history of diabetes, prior stroke or TIA, history of heart failure, chronic kidney disease and coronary artery disease. The size and color intensity of each dot correspond to the strength and direction of the association. All outcomes were assessed in N=3,817 AF patients.

Abbreviations: ALAT=Alanine aminotransferase; ANG-2=Angiopoetin-2; eGFR=estimated glomerular filtration rate; GDF-15=growth differentiation factor-15; Hs-CRP=high sensitive C-reactive protein; hsTropT=high-sensitivity troponin T; IGFBP-7= Insulin-like growth factor-binding protein-7; IL-6= Interleukin-6; NT-proBNP= N-terminal pro-B-type natriuretic peptide; OPN=Osteopontin.

Supplementary Figure 3. Associations between selected biomarkers and major bleeding, ischemic stroke and any stroke from combined Cox models of patients on oral anticoagulation (n=3,212)

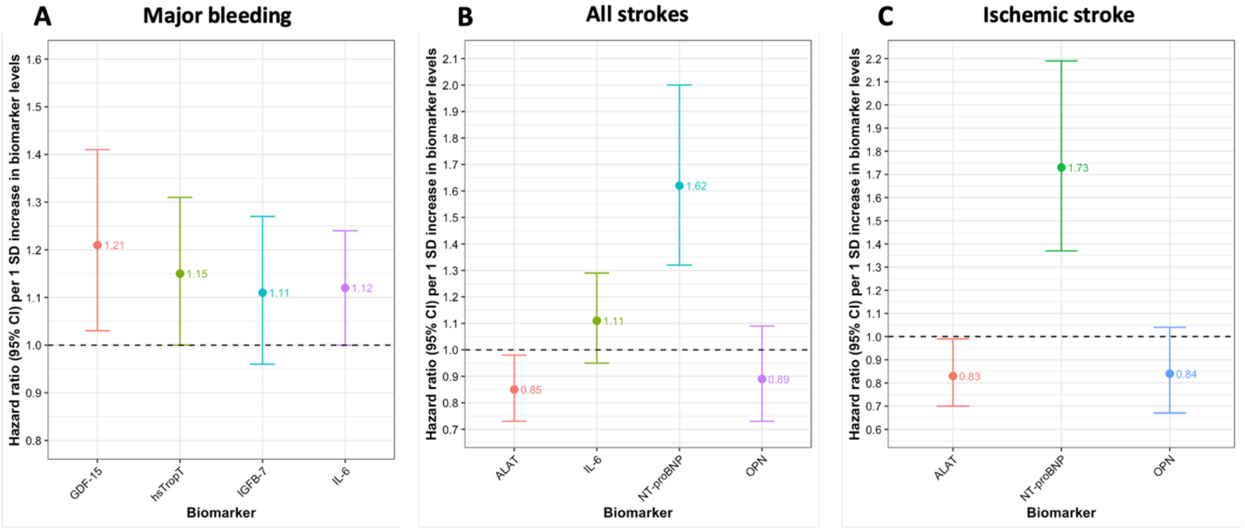

This figure shows standardized hazard ratios and 95% CIs for the associations between backward-selected biomarkers and specific adverse cardiac outcomes (major bleeding, all strokes, and ischemic stroke). Data were derived from combined multivariable Cox models in patients exclusively on oral anticoagulation (N=3,212). Panel A shows effect estimates of selected biomarkers for major bleeding. Panel B shows effect estimates of selected biomarkers for all strokes. Panel C shows effect estimates of selected biomarkers for ischemic stroke. Dots and whiskers represent hazard ratios and 95% CIs.

Abbreviations: ALAT=Alanine aminotransferase; GDF-15=growth differentiation factor-15; hsTropT=high-sensitivity troponin T; IGFBP-7= Insulin-like growth factor-binding protein-7; IL-6= Interleukin-6; NT-proBNP= N-terminal pro-B-type natriuretic peptide; OPN=Osteopontin.

Supplementary Figure 4. Relative importance of predictors from combined Cox models for major bleeding, ischemic stroke and any stroke of patients on oral anticoagulation (n=3,212)

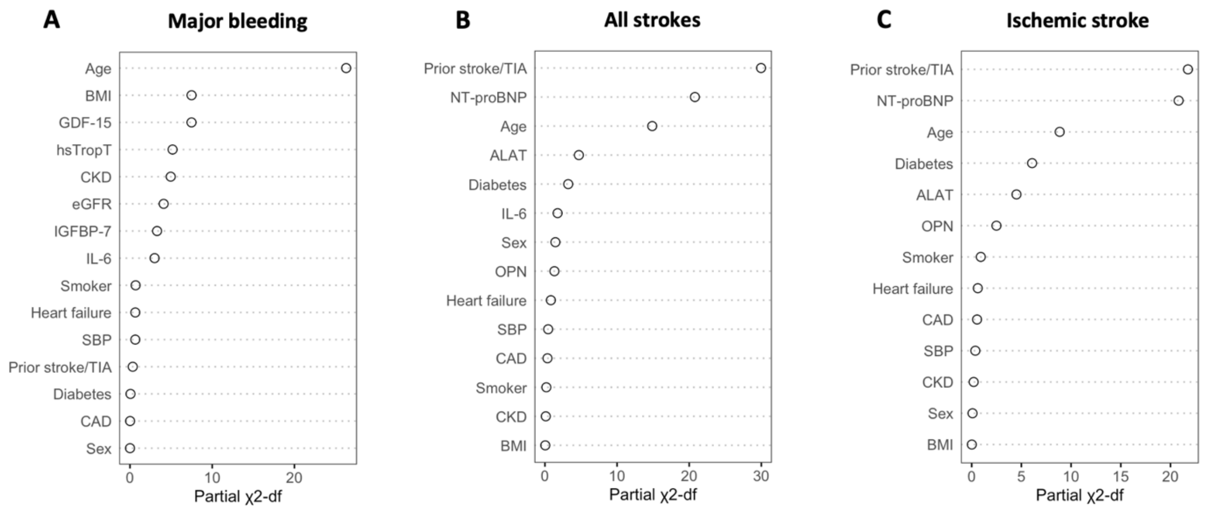

This figure shows the relative importance of each clinical variable and backward-selected biomarkers for different adverse cardiac outcomes (major bleeding, all strokes, and ischemic stroke), derived from combined multivariable Cox models. Data were derived from combined multivariable Cox models in patients exclusively on oral anticoagulation (N=3,212). Panel A shows relative importance of variables for the association with major bleeding. Panel B shows relative importance of variables for the association with all strokes. Panel C shows relative importance of variables for the association with ischemic stroke. Source data are provided as a Source Data file.

Abbreviations: ALAT=Alanine aminotransferase; ANG-2=Angiopoetin-2; BMI=body mass index; CAD=coronary artery disease; CKD=chronic kidney disease; eGFR=estimated glomerular filtration rate; GDF-15=growth differentiation factor-15; hsTropT=high-sensitivity troponin T; IGFBP-7= Insulin-like growth factor-binding protein-7; IL-6= Interleukin-6; NT-proBNP= N-terminal pro-B-type natriuretic peptide; OPN=Osteopontin; SBP=Systolic blood pressure.

Supplementary Figure 5. Discriminatory performance of clinical risk scores, biomarker-based models, and machine learning models for predicting stroke and major bleeding

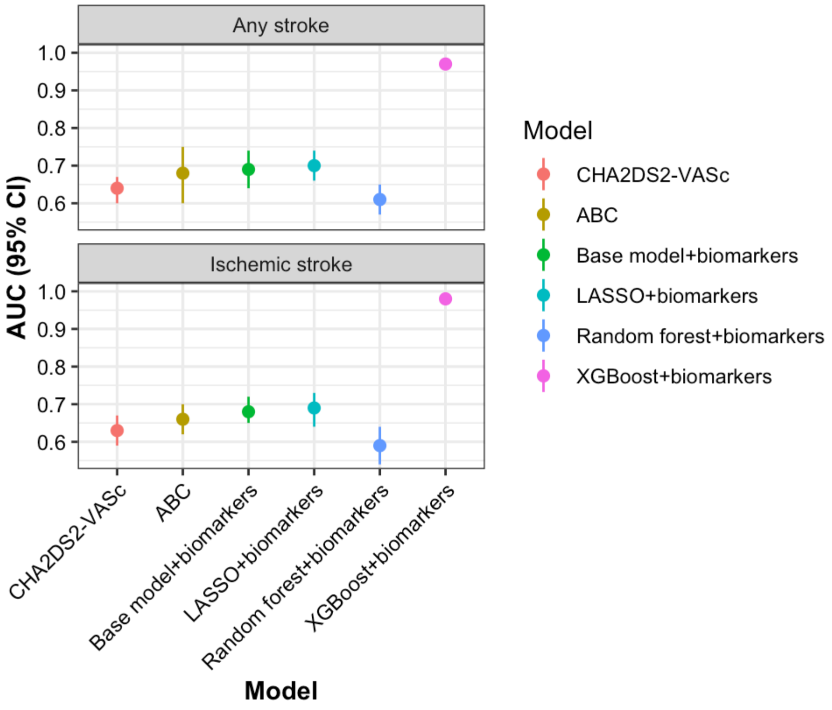

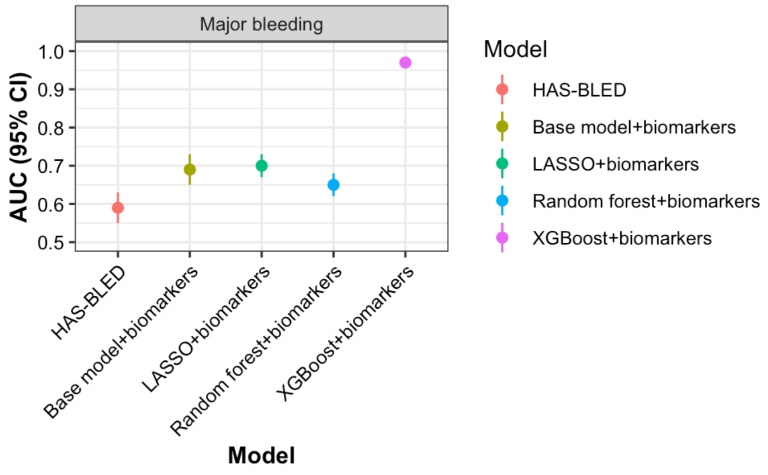

The figure shows the AUC with 95% CI for Cox and machine learning models, comparing the performance of clinical risk scores, biomarker-based models and machine learning models for different adverse cardiac outcomes (Any strokes, ischemic stroke and major bleeding). The base Cox models include age, sex, body mass index, current smoker, systolic blood pressure, history of diabetes, prior stroke or TIA, history of heart failure, chronic kidney

disease, coronary artery disease, and backward-selected biomarkers. The machine learning models include all variables listed in the Supplementary Table 16 and all biomarkers. All outcomes were assessed in N=3,817 AF patients. Dots represent AUC values and whiskers indicate 95% CIs. Source data are provided as a Source Data file.

Abbreviations: AUC=area under the curve; ABC= age, biomarkers, clinical history stroke risk score; CHA_2DS_2-VASc=Congestive heart failure, Hypertension, Age (2 points if age >75y), Diabetes, Stroke, Vascular disease, Sex category; HAS-BLED= Hypertension, Abnormal renal/liver function, Stroke, Bleeding history or predisposition, Labile international normalized ratio, Elderly (>65 years), Drugs/alcohol concomitantly.

Supplementary Figure 6. Predictive performance of Cox and machine learning models for major bleeding, ischemic stroke and any stroke with and without biomarkers of patients on oral anticoagulation (n=3,212)

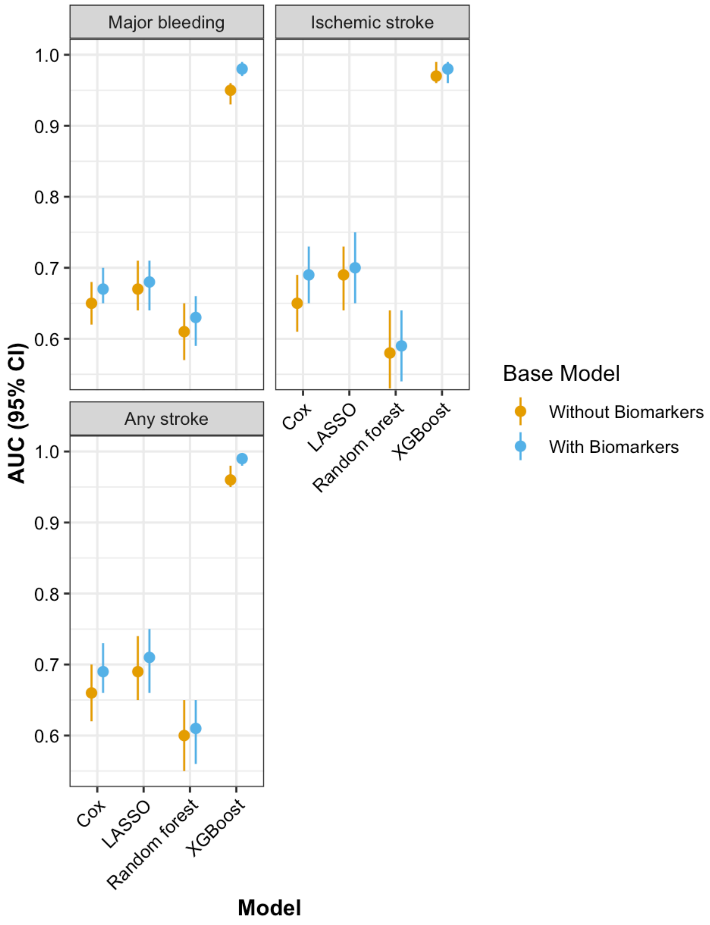

The figure shows the AUC with 95% CI for Cox and machine learning models, comparing the performance of base and base + biomarker models for different adverse cardiac outcomes (major bleeding, all strokes, and ischemic stroke). The combined Cox models include age, sex, body mass index, current smoker, systolic blood pressure, history of diabetes, prior stroke or TIA, history of heart failure, chronic kidney disease, coronary artery disease, and backward-selected biomarkers. The machine learning models include all variables listed in the Supplementary Table 16 and all biomarkers. Data were derived in patients exclusively on oral anticoagulation (N=3,212). Dots represent AUC values and whiskers indicate 95% CIs.

Abbreviations: AUC=area under the curve.

Supplementary Figure 7. Flow diagram of the study

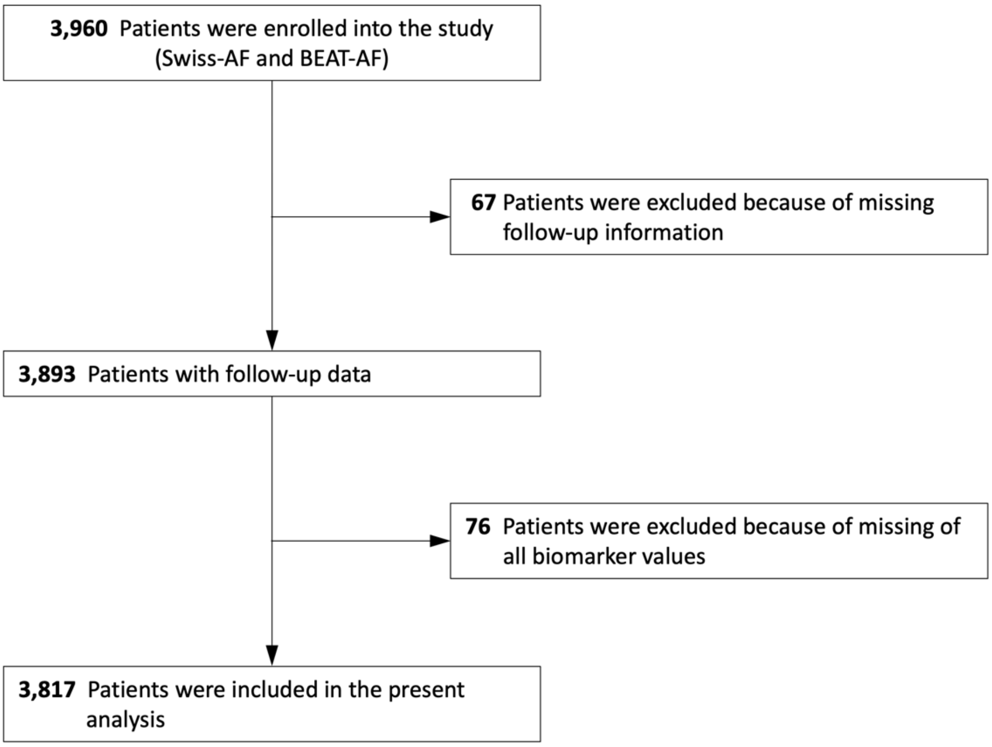

Supplementary Figure 8. Key pathophysiological pathways of the 12 selected biomarkers

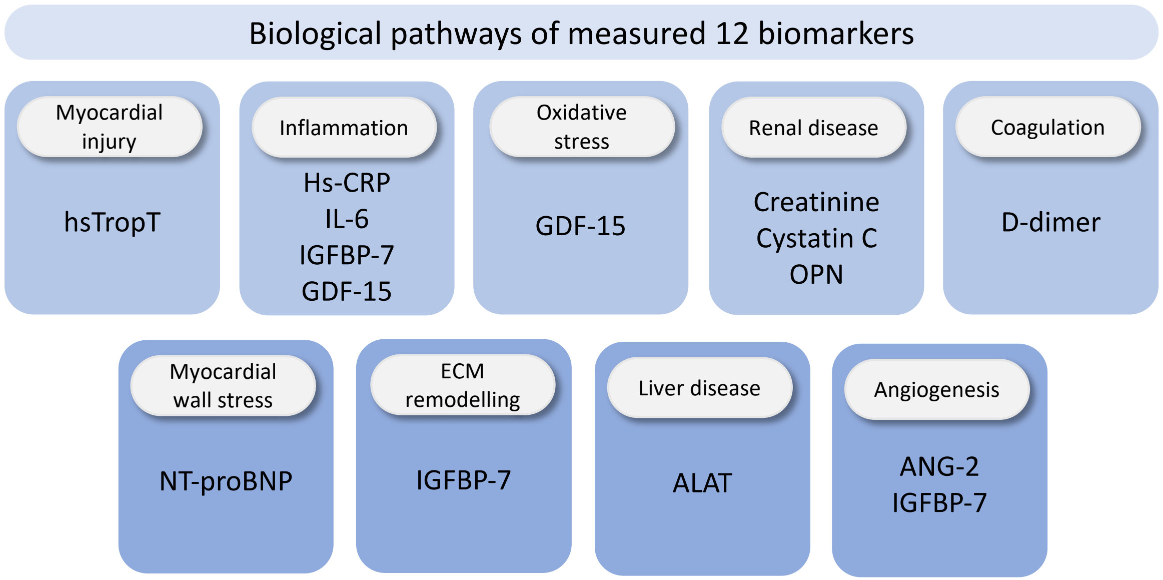

Angiopoetin-2 **(ANG-2)**, d-dimer, growth differentiation factor-15 **(GDF-15)**, insulin-like growth factor-binding protein-7 **(IGFBP-7)**, N-terminal pro-B-type natriuretic peptide **(NT-proBNP)**, high-sensitivity troponin T **(hsTropT)**, creatinine, cystatin C, osteopontin **(OPN)**, high sensitive C-reactive protein **(hs-CRP)**, interleukin-6 **(IL-6)**, and alanine aminotransferase **(ALAT)**

Supplementary Figure 9. Missing pattern and multiple imputation of biomarkers

A **B** **C**

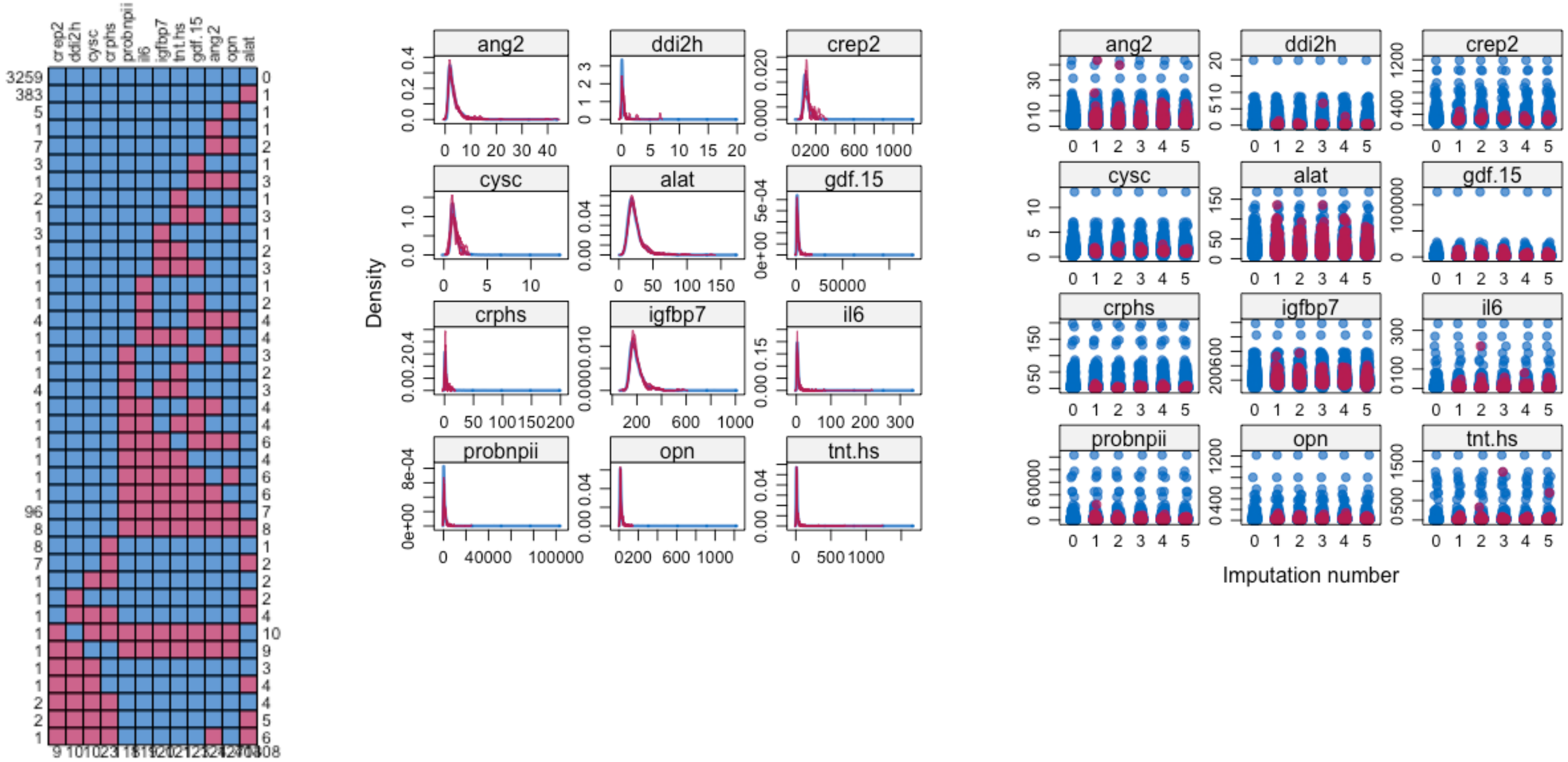

Panel A shows the distribution and extent of missing biomarker values across the dataset. The missing values are highlighted in magenta. Panel B shows the distribution of imputed values of biomarkers from multiple imputations; imputed values are highlighted in magenta. Panel C shows the distribution and variation of data points across different imputations and different biomarkers. Analyses were performed in N=3,817 AF patients.

References

1 Schulman, S. & Kearon, C. Definition of major bleeding in clinical investigations of antihemostatic medicinal products in non-surgical patients. *J Thromb Haemost* **3**, 692-694 (2005). https://doi.org:10.1111/j.1538-7836.2005.01204.x

2 Kaatz, S., Ahmad, D., Spyropoulos, A. C. & Schulman, S. Definition of clinically relevant non-major bleeding in studies of anticoagulants in atrial fibrillation and venous thromboembolic disease in non-surgical patients: communication from the SSC of the ISTH. *J Thromb Haemost* **13**, 2119-2126 (2015). https://doi.org:10.1111/jth.13140

3 Amarenco, P., Bogousslavsky, J., Caplan, L. R., Donnan, G. A. & Hennerici, M. G. Classification of stroke subtypes. *Cerebrovasc Dis* **27**, 493-501 (2009). https://doi.org:10.1159/000210432

4 Thygesen, K. *et al.* Third universal definition of myocardial infarction. *Eur Heart J* **33**, 2551-2567 (2012). https://doi.org:10.1093/eurheartj/ehs184
